# Initial Experience in Transvenous Implantation of a Left Ventricular Lead With a Novel Venogram Balloon Catheter

**DOI:** 10.3389/fcvm.2022.892122

**Published:** 2022-06-30

**Authors:** Jiangbo Duan, Dandan Yang, Jinshan He, Xuebin Li, Long Wang, Cuncao Wu, Ding Li, Feng Ze, Cuizhen Yuan, Jingliang Zhou, Xu Zhou

**Affiliations:** Department of Cardiac Electrophysiology, Peking University People’s Hospital, Beijing, China

**Keywords:** cardiac resynchronization therapy, congestive heart failure, left ventricular lead implantation, coronary sinus, venogram balloon catheter

## Abstract

**Aim:**

The most challenging and time-consuming stage of cardiac resynchronization therapy (CRT) device implantation is coronary sinus (CS) cannulation and left ventricular epicardial electrode implantation. This paper reports the initial clinical experience of CS cannulation and left ventricular lead implantation guided by a novel venogram balloon catheter (Lee’s venogram balloon catheter).

**Methods and Results:**

Consecutive patients eligible for CRT were deemed suitable for this novel venogram balloon catheter. Parameters such as left ventricular lead implantation time, procedure time, and fluoroscopy time were recorded. CS cannulation with LV lead implantation guided by Lee’s venogram balloon catheter was successful in all 5 patients, including 4 challenging cases. The total fluoroscopy and procedural durations were 5.0 ± 3.0 and 57.4 ± 12.5 min, respectively. No adverse catheter-related events occurred during the procedures.

**Conclusion:**

This initial study of an innovative venogram balloon catheter demonstrated that it greatly facilitated CS cannulation and successful LV lead placement in all patients undergoing CRT system implantation. This significantly shortened the learning curve and showed a decrease in left ventricular lead implantation time, procedure time, and fluoroscopy time.

## What’s New?

A novel venogram balloon catheter with coronary sinus shaping for implantation of left ventricular pacing lead, it is being reported for the first time.

This catheter can facilitate the coronary sinus cannulation, simplify procedures, and improve the success rate of LV lead implantation.

Several large randomized clinical trials have shown that cardiac resynchronization therapy (CRT) achieved by intraventricular and interventricular electrical mechanical desynchronization through left or biventricular pacing can effectively improve the symptoms and hemodynamic of patients with chronic congestive heart failure (CHF) and significantly reduce mortality and morbidity (1). Coronary sinus (CS) cannulation is an important stage of CRT device implantation. It is of great importance in both CS imaging and the placement of the left ventricular electrode in the appropriate region. In addition, CS cannulation is the greatest cause of procedure failure; it is also the most time-consuming and challenging aspect (2–4). Approximately 10% of attempts to place (left ventricle) LV leads are ultimately unsuccessful (5). Failure to access the CS remains the most important reason for difficult LV lead placement (4–6). Although the frequency and success of these techniques vary according to the operator, standard cannulation with a CS catheter is usually performed. Another challenge that an operator might face is that of the difficult anatomy of the coronary venous system, including sharply angulated or tortuous venous branches; due to the anatomical characteristics of the CS (4), the LV electrode is routinely implanted through the left axillary vein, and the implantation procedure becomes particularly difficult in cases that need to be implanted on the right side. As with all invasive procedures, one of the most important factors to consider in CRT implantation is radiation exposure. Shortening the fluoroscopy time is as important as the success of the procedure. Better tools and improved techniques should result in improved success rates, decreased procedure time, and decreased fluoroscopic exposure for the implanting physician. In this paper, five cases of CRT LV electrode implantation guided by a novel venogram balloon catheter (Lee’s venogram balloon catheter) *via* the right axillary vein were also reported, and the experience was summarized.

## Methods

### Population

Between October 2021 and November 2021, 5 patients with a prior pacemaker or ICD undergoing CRT upgrade were prospectively enrolled. This study complied with the Declaration of Helsinki, and the protocol was approved by the local ethics committee. Informed written consent was obtained from each patient. A comprehensive CRT pre-assessment included the New York Heart Association functional class, Minnesota Living with Heart Failure Questionnaire score, 6-min walk distance, and echocardiographic assessment of LV systolic function with 2-dimensional and 3-dimensional (3D) datasets. Patients fulfilling standard CRT criteria [New York Heart Association functional class II–IV drug refractory heart failure, left ventricle ejection fraction (LVEF) < 35%, and QRS > 150 ms] were included in the study (1).

Prior to implantation, all patients underwent clinical examination, a 12-lead electrocardiogram (ECG) recording, and routine transthoracic echocardiography (TTE). Transthoracic echocardiography was performed to assess baseline LV function and the location of infarct scars, whenever present.

## Catheter Description

**FIGURE 1:**
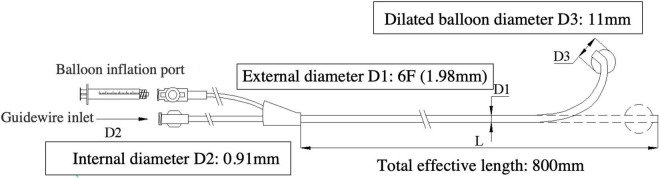
The Lee’s venogram balloon catheter (6F-11 mm-CS) with coronary sinus shaping, has an open lumen, over-the-wire design, which tracks over the guidewire and allows access to any site of the coronary vein system. Schematic diagram of the Lee’s venogram balloon with: balloon inflation port and guidewire port for access to central lumen.

Lee’s venogram balloon catheter (Figure 1), made of thermoplastic polyurethane (TPU), is a 6-Fr catheter with coronary sinus shaping. It has a central lumen, this permits one 0.035^”^ Radifocus **guidewire** or two 0.014^”^ guidewires access, transduction of pressures through a manifold, and contrast injections. There is another port for balloon occlusive venogram. The working balloon is made of a polyamide material.

### Implant Procedure

The first step involves the insertion of a 23-cm-long 9-Fr splitable introducer sheath through standard left axillary venous access (if necessary, right axillary vein can also be selected), with the tip positioned at the junction of the superior vena cava and high right atrium. Next, a 9 French (Direct™ PL 115, St. Jude) CS sheath is inserted through the sheath and placed in the right atrium, with Lee’s venogram balloon catheter (APT Medical, PRC) advanced through the lumen to the mid to lower right atrium. CS cannulation was performed by advancing a 0.035 inch Radifocus guidewire (Terumo Co., Japan) to the region of the CS ostium *via* a preformed Lee’s venogram balloon catheter and probing to locate the CS ostium. Left anterior oblique (LAO) fluoroscopic views guided Lee’s venogram balloon catheter cannulated into the CS. After successful cannulation, a Radifocus guidewire (Terumo Co., Japan) was advanced into the distal CS. Following confirmation of unrestricted guidewire movement within the distal CS, great cardiac vein (GCV) and anterior interventricular vein (AIV), Lee’s venogram balloon catheter was advanced over the wire into the CS body. The Radifocus guidewire (Terumo Co., Japan) was withdrawn, and a CS venogram (anteroposterior; left-anterior-oblique 45°) was then performed to identify branches suitable for placement of the LV lead. A suitable coronary venous branch was selectively cannulated using a runthrough-NS floppy guidewire (Terumo) passed through Lee’s venogram balloon catheter lumen, with the catheter essentially functioning as a guide for support. Once the guidewire entered the targeted vein, the guidewire was advanced deep into the distal segment of the vein to provide a track for the lead to enter into the vein and reach the desired final position. Advancing the lead while retracting the wire (the “push-pull” technique) wedges the lead tip into narrow venous segments and stabilizes the lead after withdrawing the Lee’s venogram balloon catheter. The pacing parameters were measured, and if satisfactory, the introducer sheath was carefully pulled and split outside the body.

### Statistical Analysis

Continuous variables are given as the mean ± *SD*, and categorical variables are given as percentages or frequencies.

## Results

The LV lead was successfully implanted in a target vein in all 5 patients without acute complications. Values for procedure time, fluoroscopy time, fluoroscopy dose, and contrast volume are summarized in Table 1. The mean skin-to-skin (from first incision to last stitch and dressing) procedure time was 57.4 ± 12.5 min. The LV lead implantation time was 12.6 ± 4.9 min. The fluoroscopy time was 5.0 ± 3.0 min. In one patient (20%), fluoroscopy times of < 60 s were achieved (Table 2). No adverse events occurred during the procedures or within 24 h of follow-up.

**TABLE 1:** Baseline clinical data of the five patients.

Case no.	Gender	Age	Ischemic cardiomyopathy	upgrade to CRT	CRT/CRT-D	QRS duration (ms)	LVEDD (mm)	LVEF (%)	NYHA class
1	Male	85	Yes	No	CRT	164	72	31	3
2	Male	84	Yes	No	CRT-D	177	76	28.2	4
3	Male	74	Yes	No	CRT	171	77	33.8	3
4	Female	73	No	Yes	CRT	160	67	45	3
5	Male	64	Yes	Yes	CRT	224	55	40.6	3

*CRT, cardiac resynchronization therapy; CRT-D, cardiac resynchronization therapy with defibrillator; LVEDD, left ventricular end-diastolic diameter; LVEF, left ventricular ejection fraction; NYHA, New York Heart Association.*

**TABLE 2:** Procedural details.

Case no.	LV lead implantation (min)	Procedure time (min)	Fluoroscopy time (min)	Right-sided CRT/CRT-D	Guiding catheter/sheath	Guidewire
1	8	59	0.8	No	CPS direct™ PL Peelable Outer Guide Catheter, St. Jude	Radifocus guide wire
2	10	55	3	No	CPS direct™ PL Peelable Outer Guide Catheter, St. Jude	Runthrough NS × 2
3	20	75	5	No	CPS direct™ PL Peelable Outer Guide Catheter, St. Jude	Runthrough NS × 2
4	10	40	8	Yes	CPS direct™ PL Peelable Outer Guide Catheter, St. Jude	Runthrough NS × 2
5	15	58	8	No	CPS direct™ PL Peelable Outer Guide Catheter, St. Jude	Runthrough NS × 2

*LV, left ventricle.*

## Case Description

### Case #1: Patient With Advanced HF Functional New York Heart Association Class III

**FIGURE 2:**
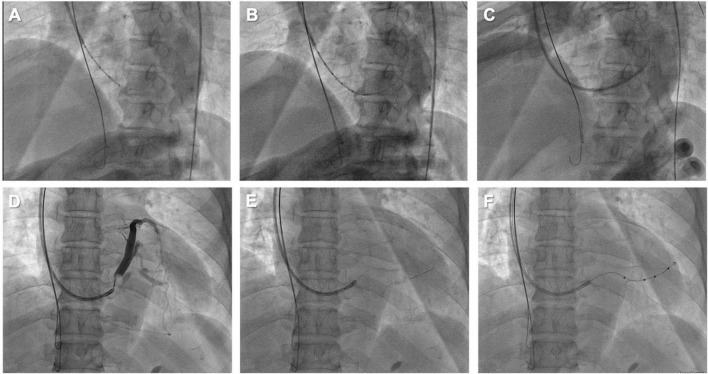
**(A)** Lee’s venogram balloon catheter advanced through the 9 French CS sheath lumen to the CS ostium. **(B)** Radifocus guidewire was successfully cannulated into the coronary sinus through Lee’s venogram balloon central lumen. **(C)** The Radifocus guidewire is advanced to the distal CS and used to advance the inner guide and 9 French CS sheath into the CS. **(D)** Lee’s venogram balloon occlusion angiogram of the CS in anteroposterior projection. **(E)** The advance runthrough-NS floppy guidewire into target vein along Lee’s venogram balloon lumen. **(F)** Once the guidewire is into the target branch, the Lee’s venogram balloon is removed and the LV lead is advanced over the guidewire.

An 85-year-old male with symptomatic HF (NYHA class III) despite optimal drug therapy, ischemic cardiomyopathy, depressed LVEF (31%), and QRS duration of 164 ms with left bundle branch abnormality morphology was referred to the Cardiology Division for further evaluation. Given the severe HF symptoms and the patient profile, CRT was desired. The LV electrode was successfully deployed *via* Lee’s venogram balloon catheter according to the above implant procedure steps (Figure 2 and Video 1). The selected epicardial pacing site was posterolateral, and the pacing threshold after anchoring the LV lead (Quartet 1458Q, St Jude Medical) was 1.3 V at 0.5 ms. The procedure time was 59 min. The LV lead implantation time was 8 min. The fluoroscopy time was 48 s. During implantation and in post-procedure follow-up, biventricular pacing was observed *via* ECG. Echo analysis indicated acute improvement in mechanical synchrony of contraction and increased LVEF.

### Case # 2: Patient With Acute Angulated Venous Branches

**FIGURE 3:**
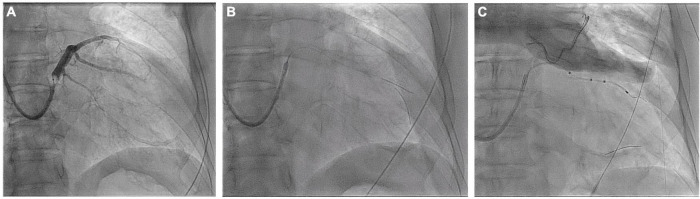
Coronary sinus venogram **(A)** and fluoroscopic images identifying the sharply angulated posterolateral branch of the coronary sinus **(B)** and the placement of the LV pacing lead using the double wire technique **(C)** (see text for details).

An 84-year-old male with ischemic cardiomyopathy, low LVEF (28%), and advanced HF functional NYHA class IV was referred to the Cardiology Division for further evaluation. The patient had three-vessel disease treated with multiple percutaneous coronary interventions; conventional angiography performed at admission showed stent patency and no further stenosis was amenable to treatment. Due to a left bundle branch block and QRS duration of 177 ms, he was treated with a cardiac resynchronization therapy with a defibrillator CRT-D device. In this patient, the desired vein had an acute angle of take-off, which made it difficult to access (Figure 3A and Video 2). Even when the vein could be subselected with a guidewire, there may have been inadequate support to track the lead over the wire without prolapse of the guidewire back into the CS. A way to approach this problem is to double-wire the acute take-off vein. As described by Chierchia (7), two 0.014^”^ runthrough-NS floppy guidewires (Terumo) are placed in the sharply angulated vein that “opens the vein,” reducing tortuosity and providing much more support. The positioning of this second guidewire against the vein’s wall effectively reduced the acuteness of the angle of the side branch, which permitted the over the-wire LV to be further advanced over the guidewire to reach a stable pacing position (Figure 3B and Video 2). Both wires were then retracted, leaving the LV lead (Quartet 1458Q, St Jude Medical) in position (Figure 3C). Pacing threshold was 0.5 V at a pulse width of 0.5 ms.

### Case 3: Patient With Acute Angulated Venous Branches Coupled With an Enlarged Right Atrium

**FIGURE 4:**
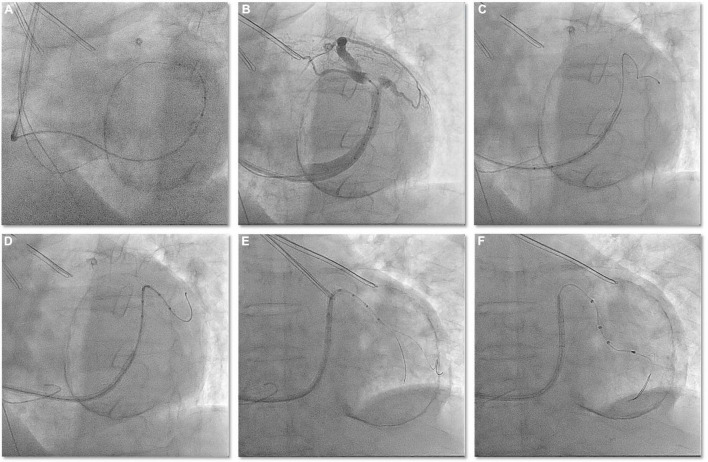
**(A)** In the case of obvious enlargement of the right atrium, the Radifocus guidewire can be easily cannulated into the CS guided by Lee’s venogram balloon. **(B)** Lee’s venogram balloon CS venography showed acute angled anteriolateral veins. **(C)** Radifocus guidewire subselectively entered the anterolateral side branch. **(D)** Lee’s venogram balloon catheter subselectively entered the anterolateral side branch along the Radifocus guidewire. **(E)** After withdraw the Radifocus guidewire, double guidewires subselectively entered the anterolateral side branch along the Lee’s venogram balloon. **(F)** LV lead is advanced into anteriolateral veins over the guidewire.

A 77-year-old man with ischemic cardiomyopathy and persistent atrial fibrillation was referred to our center because of drug refractory heart failure. At admittance, the ECG showed atrial fibrillation with a wide QRS complex of 171 ms. Transthoracic echocardiography and left ventricular angiography were performed, which showed severe left ventricular dysfunction (EF = 34%), moderate mitral valve regurgitation, and left ventricular apical aneurysm. The indication for cardiac resynchronization therapy (CRT) was made. A 9 French (CPS Direct™ PL 115, St. Jude) CS sheath was inserted in the enlarged right atrium using left axillary venous access. However, right atrial enlargement makes it difficult for conventional electrophysiological catheters to enter the CS; even if the electrophysiological catheter can enter the CS, it is difficult to provide sufficient support to introduce the CS sheath into the CS. Using Lee’s venogram balloon catheter, these difficulties can be easily overcome with the support of a Radifocus guidewire (Terumo Co., Japan) (Figure 4A and Video 3). The venogram showed a very angulated anterolateral side branch (Figure 4B and Video 3). The Radifocus guidewire was advanced in this side branch into the anterolateral branch. Lee’s venogram balloon catheter subselectively entered the anterolateral side branch along the Radifocus guidewire (Figure 4C and Video 3). Then, the Radifocus guidewire was pulled out, and a runthrough-NS floppy guidewire (Terumo) was sent into the anterolateral side branch from the lumen of Lee’s venogram balloon catheter. Negotiating the acute angle with the LV lead consistently pushed the CS guiding sheath back into the right atrium. Another runthrough-NS floppy guidewire was inserted into the anterolateral branch as a second wire to attempt to straighten the angle of the vessel (Figure 4D and Video 3). The positioning of this second guidewire against the vein’s wall effectively reduced the acuteness of the angle of the side branch and provided enough support, which permitted the over the wire LV lead (Quartet 1458Q, St Jude Medical) to be further advanced over the runthrough-NS floppy guidewire to reach a stable pacing position (Figures 4E,F). Both wires were then retracted, leaving the LV lead in position. Pacing threshold was 1.2 V at a pulse width of 0.5 ms.

### Case #4: Upgrade of Single Chamber Ventricular Pacemakers to Cardiac Resynchronization Therapy *via* the Right Axillary Vein

**FIGURE 5:**
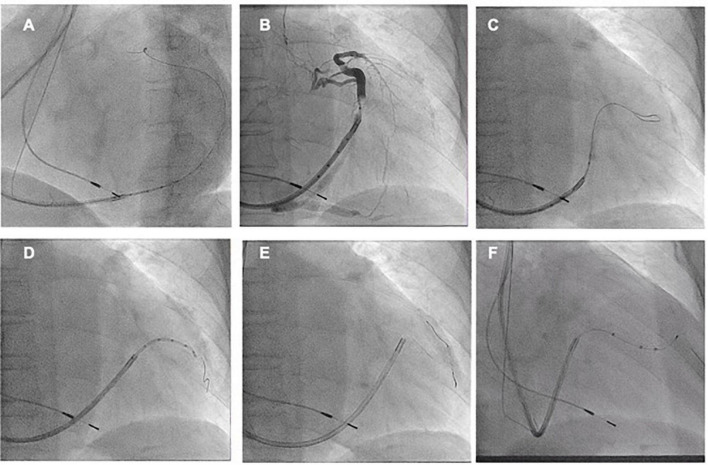
**(A)** After the right axillary venous puncture, the CS cannulation catheter (CPS Direct™ PL Peelable Outer Guide Catheter ST-JUDE) was guided through the Lee’s venogram balloon catheter to the CS ostium, and then the CS was cannulated in the left anterior oblique position by passing the Radifocus guidewire through the Lee’s venogram balloon. **(B)** Upon cannulation of the CS, the CS sheath is advanced over the Lee’s venogram balloon into the CS. Then, a coronary venous angiogram is obtained with contrast injection to delineate the venous anatomy and select the target vein (arrow). **(C,D)** After the Radifocus guidewire was subselected to enter the target vein, Lee’s venogram balloon catheter enters the target vein along the Radifocus guidewire. Lee’s venogram balloon catheter was then withdrawn. **(E,F)** After proceeding the double wires to the target branch, the LV lead was finally delivered to the lateral branch over the wire.

A 73-year-old female patient was referred to our center to upgrade a single-chamber pacemaker (implanted 4 years ago for sick sinus dysfunction) to a CRT system, following the symptoms of worsening dyspnea for 2 years, which progressed to dyspnea at rest. An ECG showed a prolonged PR interval, a left bundle branch block, and an anterior left fascicular block. The QRS interval was 160 ms in duration. A two-dimensional echocardiogram revealed a dilated LV with an end-diastolic diameter of 67 mm, an LVEF of 45%, and severe mitral regurgitation. The previously implanted right side right ventricular lead functioned well and was thus kept in place. A straight curve 9 French (CPS Direct™ PL 115, St. Jude) CS sheath was inserted in the CS using right axillary venous access. The right-sided LV lead implantation poses a unique challenge given the multiple angles the sheath must take before engaging the CS. Moreover, RA enlargement expands the subeustachian space and distorts the eustachian ridge, creating a barrier to CS entry. In this greatly enlarged RA, a Lee’s venogram balloon catheter inside a conventional guide catheter extends the reach of the system and directs a guidewire or contrast injection superiorly toward an upwardly angulated CS (Figure 5A and Video 4). Radifocus guidewire subselected lateral side branch *via* Lee’s venogram balloon catheter after CS venogram (Figure 5B and Video 4). The catheter was also subselected along the guidewire into the lateral side branch, and then the guidewire was withdrawn (Figure 5C and Video 4). To increase the supporting force, two runthrough-NS floppy guidewires (Terumo) were inserted into the lateral side branch *via* the Lee catheter. The St Jude Quartet Model 1458Q quadripolar LV lead was subsequently advanced over the wire into the lateral side branch (Figure 5D and Video 4). Both wires were then retracted, leaving the LV lead (Quartet 1458Q, St Jude Medical) in position (Figures 5E,F). The pacing threshold was 1.2 V at a pulse width of 0.5 ms.

### Case #5: Target Vessel Ostium Low and Steep Angles for Upgrading a Pre-existing DDD Pacemaker to Cardiac Resynchronization Therapy

**FIGURE 6:**
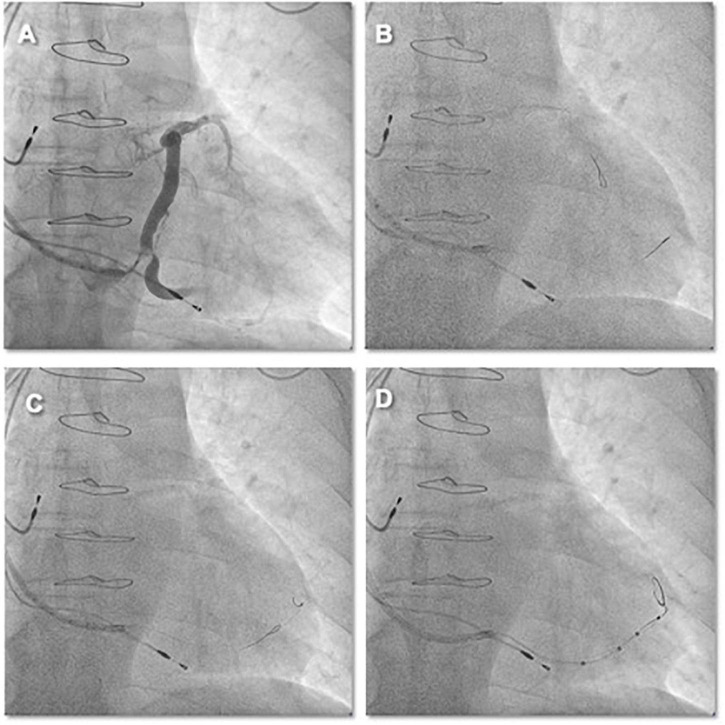
**(A)** CS venography by Lee’s venogram balloon showed that the target vessel ostium was low, sharply angled, and close to the CS ostium. Without runthrough-NS floppy guidewire anchorage, the CS sheath was easy to prolapse out of the CS. **(B)** One runthrough-NS floppy guidewire was sent to the great cardiac vein *via* Lee’s venogram balloon for anchoring. **(C,D)** The positioning of the second guidewire through Lee’s venogram balloon against the vein’s wall effectively reduced the acuteness of the angle of the side branch and this permitted the over the wire LV lead to be further advanced over the runthrough-NS floppy guidewire to reach a stable pacing position.

64-year-old male with ischemic cardiomyopathy, low LVEF (40%), and advanced HF functional NYHA class III was referred to the Cardiology Division for further evaluation. Coronary artery bypass grafting was performed 14 years ago for three-vessel coronary artery disease, and double-chamber pacemaker implantation was performed 10 years ago for sick sinus syndrome. Coronary computed tomographic angiography performed at admission showed bridge vessel patency and no further stenosis amenable to treatment. ECG at admission showed DDD pacing mode, pacing QRS duration 224 ms. Transthoracic echocardiography and left ventricular angiography were performed, which showed severe left ventricular dysfunction (LVEF = 41%) and segmental inferior wall motion abnormalities. The indication for a CRT was made. The CS venogram *via* Lee’s venogram balloon catheter showed a very angulated and tortuous target vein (inferolateral side branch) (Figure 6A and Video 5). Moreover, the target vein ostium was low, and without a guidewire anchor, the CS guiding sheath easily prolapsed back into the right atrium. One runthrough-NS floppy guidewire (Terumo) was sent through Lee’s venogram balloon catheter to the great cardiac vein for anchoring and then through Lee’s venogram balloon catheter into the second runthrough-NS floppy guidewire (Terumo) to the target vein (Figure 6B and Video 5). Since the target vein is still sharply angled with a guidewire, the guidewire in the great cardiac vein is also delivered into the target vein. Using this “double wire technique,” tortuosities in the target branch are straightened, allowing uninhibited lead advancement (Figure 6C and Video 5). The “double wire technique” reduces the abruptness of the angle of sharply angulated CS side branches by placing two wires in the target vessel. By keeping both wires in the side branch, a wire that is positioned against the wall of the vein can decrease the sharp angle, permitting the over the wire LV lead (Quartet 1458Q, St Jude Medical) to be finally delivered to the inferolateral branch over the other guidewire (Figure 6D and Video 5).

### Complications

No adverse catheter-related events occurred during the procedures. No reoperation due to LV leads capture loss, phrenic nerve pacing, or infection occurred during follow-up.

## Discussion

In this article, we demonstrate the feasibility and safety of using Lee’s venogram balloon catheter to guide CS cannulation and St Jude Quartet Model 1458Q quadripolar LV lead implantation. We demonstrated this in five patients who had standard indications for CRT, in whom this catheter was used as a first-line tool.

Our preliminary experience shows that Lee’s venogram balloon catheter has the following advantages. First, Lee’s venogram balloon catheter shape was compatible with patient’s coronary sinus location, so this catheter facilitates CS cannulation. Second, under the guidance of Radifocus guidewire, Lee’s venogram balloon catheter can be safely advanced to the distal end of great cardiac vein to avoid dissection. Once the Lee’s catheter and the Radifocus guidewire reach the distal end of the coronary sinus, they can provide sufficient support to facilitate the delivery of the CS sheath, especially in cases where CS cannulation is difficult, such as patients with right atrial enlargement or patients with right-sided CRT implantation. Third, compared with the conventional catheters, Lee’s venogram balloon catheter can integrate three operation steps: coronary sinus cannulation, coronary sinus venography, and guidewire into the target vessel, which simplifies the procedure and process and shortens the procedure time. Further combination of the double-wire technique can effectively overcome the difficulty of sharply angulated CS branches during left ventricular electrode implantation. Finally, it is no longer more expensive than traditional EP catheters and is comparably priced.

The use of Lee’s venogram balloon catheter-guided CS cannulation along with guidewire and balloon hence provides a multiplicity of features within a single tool. It is possible that the novel venogram balloon catheter may shorten the learning curve for each individual operator. Larger studies are required for a comparison with standard techniques.

## Conclusion

In this article, we describe a novel Lee’s venogram balloon catheter guiding CS cannulation and LV lead implantation. This catheter involves utilizing a CS venogram balloon with a central lumen that can provide contrast injection and allow access to the CS over the guidewire. Once familiar, this catheter may provide a less complicated strategy that improves success rates, decreases procedure time, and decreases fluoroscopic exposure for the implanting physician.

## Data Availability Statement

The original contributions presented in the study are included in the article/supplementary material, further inquiries can be directed to the corresponding author/s.

## Ethics Statement

The studies involving human participants were reviewed and approved by the Ethics Committee of Peking University. The patients/participants provided their written informed consent to participate in this case study. Written informed consent was obtained from the individual(s) for the publication of any potentially identifiable images or data included in this article.

## Author Contributions

JD and DY: conceptualization, methodology, software, investigation, formal analysis, writing—original draft, visualization, and writing—review and editing. JH: data curation and writing—original draft. LW: visualization and investigation. CW, DL, FZ, and CY: resources, supervision, software, and validation. XL: conceptualization, funding acquisition, resources, supervision, and writing—review and editing. All authors contributed to the article and approved the submitted version.

## Conflict of Interest

The authors declare that the research was conducted in the absence of any commercial or financial relationships that could be construed as a potential conflict of interest.

## Publisher’s Note

All claims expressed in this article are solely those of the authors and do not necessarily represent those of their affiliated organizations, or those of the publisher, the editors and the reviewers. Any product that may be evaluated in this article, or claim that may be made by its manufacturer, is not guaranteed or endorsed by the publisher.
